# Supplementary material for: Visible and near-infrared radiation may be transmitted or absorbed differently by beetle elytra according to habitat preference
Source: PeerJ. 2019 Nov 25;7:e8104. doi: 10.7717/peerj.8104 (PMC6882417; doi:10.7717/peerj.8104)
Supplement: Supplemental Information 1 [file peerj-07-8104-s001.docx]

| **ID** | **Treatment** | **Elytra side** | **SPP** | **Specimen** | **Body weight (mg)** | **Elyton area (mm^2^)** | **Elytra thickness (mm)** | **Dark spots (in %)** | **Mean for 200-385 nm** | **Mean for 390-745 nm** | **Mean for 750-1400 nm** |
| --- | --- | --- | --- | --- | --- | --- | --- | --- | --- | --- | --- |
| 1 | Reflectance | Dorsal | *O. coenobita* | 1 | 71 | 10.38 | 0.097 | 3.542 | 0.485 | 2.900 | 9.015 |
| 2 | Reflectance | Dorsal | *O. coenobita* | 2 | 41 | 7.27 | 0.074 | 6.355 | 0.323 | 1.536 | 4.436 |
| 3 | Reflectance | Dorsal | *O. coenobita* | 3 | 53 | 8.20 | 0.081 | 2.402 | 0.236 | 3.534 | 9.874 |
| 4 | Reflectance | Dorsal | *O. coenobita* | 4 | 69 | 9.84 | 0.087 | 1.821 | 0.212 | 2.051 | 6.424 |
| 5 | Reflectance | Dorsal | *O. coenobita* | 5 | 60 | 8.64 | 0.079 | 2.979 | 0.184 | 1.860 | 6.465 |
| 6 | Reflectance | Dorsal | *O. coenobita* | 6 | 56 | 10.87 | 0.076 | 1.808 | 0.317 | 2.758 | 10.175 |
| 7 | Reflectance | Dorsal | *O. coenobita* | 7 | 46 | 9.00 | 0.090 | 4.109 | 0.267 | 2.649 | 8.939 |
| 8 | Reflectance | Dorsal | *O. coenobita* | 8 | 47 | 8.64 | 0.087 | 4.485 | 0.258 | 2.773 | 9.800 |
| 9 | Reflectance | Dorsal | *O. coenobita* | 9 | 55 | 8.64 | 0.097 | 2.298 | 0.192 | 2.436 | 8.780 |
| 10 | Reflectance | Dorsal | *O. coenobita* | 10 | 29 | 6.43 | 0.076 | 6.995 | 0.256 | 2.191 | 7.275 |
| 41 | Transmittance | Dorsal | *O. coenobita* | 1 | 71 | 10.38 | 0.097 | 3.542 | 0.006 | 13.351 | 38.798 |
| 42 | Transmittance | Dorsal | *O. coenobita* | 2 | 41 | 7.27 | 0.074 | 6.355 | 0.214 | 17.636 | 45.876 |
| 43 | Transmittance | Dorsal | *O. coenobita* | 3 | 53 | 8.20 | 0.081 | 2.402 | 0.016 | 17.631 | 47.570 |
| 44 | Transmittance | Dorsal | *O. coenobita* | 4 | 69 | 9.84 | 0.087 | 1.821 | 1.563 | 17.824 | 51.312 |
| 45 | Transmittance | Dorsal | *O. coenobita* | 5 | 60 | 8.64 | 0.079 | 2.979 | 0.709 | 16.553 | 50.155 |
| 46 | Transmittance | Dorsal | *O. coenobita* | 6 | 56 | 10.87 | 0.076 | 1.808 | 0.137 | 17.427 | 50.834 |
| 47 | Transmittance | Dorsal | *O. coenobita* | 7 | 46 | 9.00 | 0.090 | 4.109 | 0.670 | 20.425 | 51.428 |
| 48 | Transmittance | Dorsal | *O. coenobita* | 8 | 47 | 8.64 | 0.087 | 4.485 | 2.824 | 15.737 | 44.221 |
| 49 | Transmittance | Dorsal | *O. coenobita* | 9 | 55 | 8.64 | 0.097 | 2.298 | 0.562 | 12.948 | 41.225 |
| 50 | Transmittance | Dorsal | *O. coenobita* | 10 | 29 | 6.43 | 0.076 | 6.995 | 4.647 | 20.413 | 42.809 |
| 81 | Absorbance | Dorsal | *O. coenobita* | 1 | 71 | 10.38 | 0.097 | 3.542 | 99.508 | 83.748 | 52.187 |
| 82 | Absorbance | Dorsal | *O. coenobita* | 2 | 41 | 7.27 | 0.074 | 6.355 | 99.464 | 80.828 | 49.688 |
| 83 | Absorbance | Dorsal | *O. coenobita* | 3 | 53 | 8.20 | 0.081 | 2.402 | 99.748 | 78.835 | 42.556 |
| 84 | Absorbance | Dorsal | *O. coenobita* | 4 | 69 | 9.84 | 0.087 | 1.821 | 98.225 | 80.125 | 42.265 |
| 85 | Absorbance | Dorsal | *O. coenobita* | 5 | 60 | 8.64 | 0.079 | 2.979 | 99.107 | 81.587 | 43.380 |
| 86 | Absorbance | Dorsal | *O. coenobita* | 6 | 56 | 10.87 | 0.076 | 1.808 | 99.546 | 79.815 | 38.991 |
| 87 | Absorbance | Dorsal | *O. coenobita* | 7 | 46 | 9.00 | 0.090 | 4.109 | 99.063 | 76.926 | 39.633 |
| 88 | Absorbance | Dorsal | *O. coenobita* | 8 | 47 | 8.64 | 0.087 | 4.485 | 96.918 | 81.490 | 45.978 |
| 89 | Absorbance | Dorsal | *O. coenobita* | 9 | 55 | 8.64 | 0.097 | 2.298 | 99.246 | 84.616 | 49.996 |
| 90 | Absorbance | Dorsal | *O. coenobita* | 10 | 29 | 6.43 | 0.076 | 6.995 | 95.097 | 77.395 | 49.916 |
| 101 | Absorbance | Ventral | *O. coenobita* | 1 | 71 | 10.38 | 0.097 | 3.542 |  |  | 43.510 |
| 102 | Absorbance | Ventral | *O. coenobita* | 2 | 41 | 7.27 | 0.074 | 6.355 |  |  | 45.109 |
| 103 | Absorbance | Ventral | *O. coenobita* | 3 | 53 | 8.20 | 0.081 | 2.402 |  |  | 37.457 |
| 104 | Absorbance | Ventral | *O. coenobita* | 4 | 69 | 9.84 | 0.087 | 1.821 |  |  | 40.158 |
| 105 | Absorbance | Ventral | *O. coenobita* | 5 | 60 | 8.64 | 0.079 | 2.979 |  |  | 36.384 |
| 106 | Absorbance | Ventral | *O. coenobita* | 6 | 56 | 10.87 | 0.076 | 1.808 |  |  | 31.545 |
| 107 | Absorbance | Ventral | *O. coenobita* | 7 | 46 | 9.00 | 0.090 | 4.109 |  |  | 30.604 |
| 108 | Absorbance | Ventral | *O. coenobita* | 8 | 47 | 8.64 | 0.087 | 4.485 |  |  | 38.248 |
| 109 | Absorbance | Ventral | *O. coenobita* | 9 | 55 | 8.64 | 0.097 | 2.298 |  |  | 41.705 |
| 110 | Absorbance | Ventral | *O. coenobita* | 10 | 29 | 6.43 | 0.076 | 6.995 |  |  | 34.127 |
| 21 | Reflectance | Ventral | *O. coenobita* | 1 | 71 | 10.38 | 0.097 | 3.542 |  |  | 8.973 |
| 22 | Reflectance | Ventral | *O. coenobita* | 2 | 41 | 7.27 | 0.074 | 6.355 |  |  | 4.995 |
| 23 | Reflectance | Ventral | *O. coenobita* | 3 | 53 | 8.20 | 0.081 | 2.402 |  |  | 9.570 |
| 24 | Reflectance | Ventral | *O. coenobita* | 4 | 69 | 9.84 | 0.087 | 1.821 |  |  | 5.816 |
| 25 | Reflectance | Ventral | *O. coenobita* | 5 | 60 | 8.64 | 0.079 | 2.979 |  |  | 6.751 |
| 26 | Reflectance | Ventral | *O. coenobita* | 6 | 56 | 10.87 | 0.076 | 1.808 |  |  | 10.097 |
| 27 | Reflectance | Ventral | *O. coenobita* | 7 | 46 | 9.00 | 0.090 | 4.109 |  |  | 11.260 |
| 28 | Reflectance | Ventral | *O. coenobita* | 8 | 47 | 8.64 | 0.087 | 4.485 |  |  | 8.978 |
| 29 | Reflectance | Ventral | *O. coenobita* | 9 | 55 | 8.64 | 0.097 | 2.298 |  |  | 8.605 |
| 30 | Reflectance | Ventral | *O. coenobita* | 10 | 29 | 6.43 | 0.076 | 6.995 |  |  | 5.771 |
| 61 | Transmittance | Ventral | *O. coenobita* | 1 | 71 | 10.38 | 0.097 | 3.542 |  |  | 47.517 |
| 62 | Transmittance | Ventral | *O. coenobita* | 2 | 41 | 7.27 | 0.074 | 6.355 |  |  | 49.897 |
| 63 | Transmittance | Ventral | *O. coenobita* | 3 | 53 | 8.20 | 0.081 | 2.402 |  |  | 52.973 |
| 64 | Transmittance | Ventral | *O. coenobita* | 4 | 69 | 9.84 | 0.087 | 1.821 |  |  | 54.026 |
| 65 | Transmittance | Ventral | *O. coenobita* | 5 | 60 | 8.64 | 0.079 | 2.979 |  |  | 56.865 |
| 66 | Transmittance | Ventral | *O. coenobita* | 6 | 56 | 10.87 | 0.076 | 1.808 |  |  | 58.358 |
| 67 | Transmittance | Ventral | *O. coenobita* | 7 | 46 | 9.00 | 0.090 | 4.109 |  |  | 58.137 |
| 68 | Transmittance | Ventral | *O. coenobita* | 8 | 47 | 8.64 | 0.087 | 4.485 |  |  | 52.774 |
| 69 | Transmittance | Ventral | *O. coenobita* | 9 | 55 | 8.64 | 0.097 | 2.298 |  |  | 49.689 |
| 70 | Transmittance | Ventral | *O. coenobita* | 10 | 29 | 6.43 | 0.076 | 6.995 |  |  | 60.103 |
| 11 | Reflectance | Dorsal | *O. medius* | 1 | 146 | 15.31 | 0.104 | 30.363 | 0.323 | 2.512 | 9.673 |
| 12 | Reflectance | Dorsal | *O. medius* | 2 | 122 | 13.62 | 0.106 | 30.793 | 0.339 | 1.321 | 4.578 |
| 13 | Reflectance | Dorsal | *O. medius* | 3 | 90 | 11.61 | 0.085 | 20.267 | 0.274 | 2.173 | 6.918 |
| 14 | Reflectance | Dorsal | *O. medius* | 4 | 166 | 16.25 | 0.124 | 23.494 | 0.842 | 3.135 | 10.259 |
| 15 | Reflectance | Dorsal | *O. medius* | 5 | 80 | 11.49 | 0.089 | 29.760 | 0.122 | 2.656 | 11.098 |
| 16 | Reflectance | Dorsal | *O. medius* | 6 | 90 | 12.59 | 0.100 | 21.070 | 0.654 | 2.838 | 9.969 |
| 17 | Reflectance | Dorsal | *O. medius* | 7 | 62 | 10.58 | 0.082 | 24.956 | 0.475 | 2.327 | 8.159 |
| 18 | Reflectance | Dorsal | *O. medius* | 8 | 69 | 11.02 | 0.087 | 14.705 | 0.661 | 4.562 | 11.538 |
| 19 | Reflectance | Dorsal | *O. medius* | 9 | 107 | 14.11 | 0.115 | 22.635 | 0.766 | 3.270 | 10.894 |
| 20 | Reflectance | Dorsal | *O. medius* | 10 | 117 | 14.54 | 0.104 | 27.902 | 0.833 | 3.521 | 12.324 |
| 51 | Transmittance | Dorsal | *O. medius* | 1 | 146 | 15.31 | 0.104 | 30.363 | 0.072 | 7.080 | 36.505 |
| 52 | Transmittance | Dorsal | *O. medius* | 2 | 122 | 13.62 | 0.106 | 30.793 | 0.050 | 8.348 | 37.944 |
| 53 | Transmittance | Dorsal | *O. medius* | 3 | 90 | 11.61 | 0.085 | 20.267 | 2.527 | 18.770 | 52.259 |
| 54 | Transmittance | Dorsal | *O. medius* | 4 | 166 | 16.25 | 0.124 | 23.494 | 5.738 | 13.499 | 35.755 |
| 55 | Transmittance | Dorsal | *O. medius* | 5 | 80 | 11.49 | 0.089 | 29.760 | 5.507 | 17.455 | 49.245 |
| 56 | Transmittance | Dorsal | *O. medius* | 6 | 90 | 12.59 | 0.100 | 21.070 | 0.597 | 12.926 | 40.774 |
| 57 | Transmittance | Dorsal | *O. medius* | 7 | 62 | 10.58 | 0.082 | 24.956 | 1.466 | 11.972 | 44.640 |
| 58 | Transmittance | Dorsal | *O. medius* | 8 | 69 | 11.02 | 0.087 | 14.705 | 4.330 | 25.934 | 52.427 |
| 59 | Transmittance | Dorsal | *O. medius* | 9 | 107 | 14.11 | 0.115 | 22.635 | 0.573 | 14.560 | 47.800 |
| 60 | Transmittance | Dorsal | *O. medius* | 10 | 117 | 14.54 | 0.104 | 27.902 | 0.859 | 9.150 | 38.023 |
| 91 | Absorbance | Dorsal | *O. medius* | 1 | 146 | 15.31 | 0.104 | 30.363 | 99.605 | 90.408 | 53.822 |
| 92 | Absorbance | Dorsal | *O. medius* | 2 | 122 | 13.62 | 0.106 | 30.793 | 99.611 | 90.331 | 57.478 |
| 93 | Absorbance | Dorsal | *O. medius* | 3 | 90 | 11.61 | 0.085 | 20.267 | 97.199 | 79.057 | 40.823 |
| 94 | Absorbance | Dorsal | *O. medius* | 4 | 166 | 16.25 | 0.124 | 23.494 | 93.420 | 83.366 | 53.986 |
| 95 | Absorbance | Dorsal | *O. medius* | 5 | 80 | 11.49 | 0.089 | 29.760 | 94.372 | 79.889 | 39.657 |
| 96 | Absorbance | Dorsal | *O. medius* | 6 | 90 | 12.59 | 0.100 | 21.070 | 98.750 | 84.236 | 49.257 |
| 97 | Absorbance | Dorsal | *O. medius* | 7 | 62 | 10.58 | 0.082 | 24.956 | 98.059 | 85.701 | 47.201 |
| 98 | Absorbance | Dorsal | *O. medius* | 8 | 69 | 11.02 | 0.087 | 14.705 | 95.009 | 69.505 | 36.036 |
| 99 | Absorbance | Dorsal | *O. medius* | 9 | 107 | 14.11 | 0.115 | 22.635 | 98.661 | 82.170 | 41.306 |
| 100 | Absorbance | Dorsal | *O. medius* | 10 | 117 | 14.54 | 0.104 | 27.902 | 98.308 | 87.329 | 49.653 |
| 111 | Absorbance | Ventral | *O. medius* | 1 | 146 | 15.31 | 0.104 | 30.363 |  |  | 52.211 |
| 112 | Absorbance | Ventral | *O. medius* | 2 | 122 | 13.62 | 0.106 | 30.793 |  |  | 53.787 |
| 113 | Absorbance | Ventral | *O. medius* | 3 | 90 | 11.61 | 0.085 | 20.267 |  |  | 42.510 |
| 114 | Absorbance | Ventral | *O. medius* | 4 | 166 | 16.25 | 0.124 | 23.494 |  |  | 46.692 |
| 115 | Absorbance | Ventral | *O. medius* | 5 | 80 | 11.49 | 0.089 | 29.760 |  |  | 34.749 |
| 116 | Absorbance | Ventral | *O. medius* | 6 | 90 | 12.59 | 0.100 | 21.070 |  |  | 35.074 |
| 117 | Absorbance | Ventral | *O. medius* | 7 | 62 | 10.58 | 0.082 | 24.956 |  |  | 38.434 |
| 118 | Absorbance | Ventral | *O. medius* | 8 | 69 | 11.02 | 0.087 | 14.705 |  |  | 30.967 |
| 119 | Absorbance | Ventral | *O. medius* | 9 | 107 | 14.11 | 0.115 | 22.635 |  |  | 47.612 |
| 120 | Absorbance | Ventral | *O. medius* | 10 | 117 | 14.54 | 0.104 | 27.902 |  |  | 43.245 |
| 31 | Reflectance | Ventral | *O. medius* | 1 | 146 | 15.31 | 0.104 | 30.363 |  |  | 7.904 |
| 32 | Reflectance | Ventral | *O. medius* | 2 | 122 | 13.62 | 0.106 | 30.793 |  |  | 6.886 |
| 33 | Reflectance | Ventral | *O. medius* | 3 | 90 | 11.61 | 0.085 | 20.267 |  |  | 7.925 |
| 34 | Reflectance | Ventral | *O. medius* | 4 | 166 | 16.25 | 0.124 | 23.494 |  |  | 12.594 |
| 35 | Reflectance | Ventral | *O. medius* | 5 | 80 | 11.49 | 0.089 | 29.760 |  |  | 12.448 |
| 36 | Reflectance | Ventral | *O. medius* | 6 | 90 | 12.59 | 0.100 | 21.070 |  |  | 9.527 |
| 37 | Reflectance | Ventral | *O. medius* | 7 | 62 | 10.58 | 0.082 | 24.956 |  |  | 7.940 |
| 38 | Reflectance | Ventral | *O. medius* | 8 | 69 | 11.02 | 0.087 | 14.705 |  |  | 10.174 |
| 39 | Reflectance | Ventral | *O. medius* | 9 | 107 | 14.11 | 0.115 | 22.635 |  |  | 12.004 |
| 40 | Reflectance | Ventral | *O. medius* | 10 | 117 | 14.54 | 0.104 | 27.902 |  |  | 12.605 |
| 71 | Transmittance | Ventral | *O. medius* | 1 | 146 | 15.31 | 0.104 | 30.363 |  |  | 39.885 |
| 72 | Transmittance | Ventral | *O. medius* | 2 | 122 | 13.62 | 0.106 | 30.793 |  |  | 39.327 |
| 73 | Transmittance | Ventral | *O. medius* | 3 | 90 | 11.61 | 0.085 | 20.267 |  |  | 49.565 |
| 74 | Transmittance | Ventral | *O. medius* | 4 | 166 | 16.25 | 0.124 | 23.494 |  |  | 40.714 |
| 75 | Transmittance | Ventral | *O. medius* | 5 | 80 | 11.49 | 0.089 | 29.760 |  |  | 52.803 |
| 76 | Transmittance | Ventral | *O. medius* | 6 | 90 | 12.59 | 0.100 | 21.070 |  |  | 55.399 |
| 77 | Transmittance | Ventral | *O. medius* | 7 | 62 | 10.58 | 0.082 | 24.956 |  |  | 53.626 |
| 78 | Transmittance | Ventral | *O. medius* | 8 | 69 | 11.02 | 0.087 | 14.705 |  |  | 58.859 |
| 79 | Transmittance | Ventral | *O. medius* | 9 | 107 | 14.11 | 0.115 | 22.635 |  |  | 40.385 |
| 80 | Transmittance | Ventral | *O. medius* | 10 | 117 | 14.54 | 0.104 | 27.902 |  |  | 44.150 |
